# Biomethane Yield, Physicochemical Structures, and Microbial Community Characteristics of Corn Stover Pretreated by Urea Combined with Mild Temperature Hydrotherm

**DOI:** 10.3390/polym13132207

**Published:** 2021-07-03

**Authors:** Yao Lu, Hairong Yuan, Xiaoyu Zuo, Yanqing Chang, Xiujin Li

**Affiliations:** 1 Beijing Engineering Center for Pollution Control and Resource Recovery, Beijing University of Chemical Technology, Beijing 100029, China; luyao@mail.buct.edu.cn (Y.L.); yuanhairong75@163.com (H.Y.); zuoxy@mail.buct.edu.cn (X.Z.); 2 WELLE Environmental Group Co., Ltd., Changzhou 213125, China; changyanqing@wellegroup.com

**Keywords:** anaerobic digestion, pretreatment, urea, hydrothermal, whole slurry, microbial community

## Abstract

The corn stover (CS)’s compact structure makes it challenging for microorganisms to use in anaerobic digestion (AD). Therefore, improving CS biodegradability has become a key focus in AD studies. Methods are being targeted at the pretreatment of CS, combining advanced urea with mild temperature hydrotherm pretreatment to study its effect on promoting the AD process of CS. The biomethane yield, physicochemical structure, and microbial community characteristics were investigated. CS samples were assigned into groups differed by a range of pretreatment times (from 24 to 96 h) and set at a temperature of 50 °C with a 2% urea addition. Results revealed that the 72-h group obtained the highest biomethane yield of 205 mL/g VS^−1^, volatile solid (VS) and total solid (TS) removal rates of 69.3% and 47.7%, which were 36.7%, 25.3% and 27.5% higher than those of untreated one, respectively. After conducting several analyses, results confirmed the pretreatment as a method for altering CS microstructures benefits biomethane production. The most resounding differences between pretreated and untreated groups were observed within a microbial community, an integral factor for improved AD performance. This study serves to confirm that this specific pretreatment is an effective method for enhancing biomethane production in CS.

## 1. Introduction

China is the second-largest energy consumer in the world. With total energy consumption on a steady rise as their economy continues to develop. According to statistics, China’s total energy consumption reached 4870 million tonnes in 2019 based on standard coal [1]. Meanwhile, China is also one of the largest agricultural export countries, responsible for a considerable portion of global crop production. Corn is one of three major crops in China, with corn production reaching 260.8 million tonnes in 2019. This production directly results in a sizeable 344.2 million tonnes of corn stover (CS), consisting of the leaves, stalks and cobs of the plant leftover after harvest [1]. Although CS is frequently reused in various ways, large amounts are either abandoned or burned in open fields, contributing to resource waste and polluting the air [2]. 

Only a few technologies have been developed for CS treatment and reuse, including animal feed, fertiliser or direct combustion to generate electricity [3,4,5]. One of these technologies, anaerobic digestion (AD), has garnered significant attention in recent years. When compared to other available technologies, AD has several advantages, such as mitigating waste pollution, producing clean bioenergy and reuse as organic fertiliser. Approximately 70 billion m^3^ of biomethane can be produced annually if all the CS in China were converted through AD technology. This amount would account for 22.8% of total natural gas consumption—or 43.4% of all imported natural gas—in China for 2019 [1]. In this way, AD technology can play an important role in providing clean bioenergy for China.

The main components of CS are cellulose, hemicellulose and lignin, mutually crosslinked to form a complex three-dimensional (3D) structure. This structure is highly resistant to degradation through anaerobic microbes, leading to long digestion time and low biomethane yields. AD technology is not being used widely for industrial-scale bioconversion of lignocellulosic substrates in China. The key challenge is how to improve CS biodegradability to achieve high-efficiency anaerobic digestion (AD). 

Pretreatment before the AD process is one of the most effective approaches for improving the biodegradability of lignocellulosic feedstocks such as CS, with numerous studies previously conducted in this area. Generally, pretreatment can be classified into physical, chemical, biological pretreatment, and the combination of them [6]. No matter what method is applied, the basic principle is to destroy the chemical structure, decrease cellulose crystallinity, increase effective surface area and decompose the lignin element. Among these methods, chemical pretreatment is regarded as most effective and is consequently more widely applied. Chemicals commonly used include acids (H_2_SO_4_, HPO_4_), alkali (NaOH, KOH, NH_3_·H_2_O, CO(NH_2_)_2_), oxidant agents (H_2_O_2_, O_3_), organic solvents (ethyl alcohol, methyl alcohol, acetone) and ionic liquids [7,8]. The alkaline pretreatment has been more widely studied with the most proven and trustworthy results. Zheng et al. [9] reported a 72.9% gas production increase through NaOH-pretreated CS. He et al. [10] supported this by reporting a 58.1% improvement on biogas yields from rice straw following a solid-state NaOH pretreatment. The study further found that ester bonds between lignin and hemicellulose were destroyed, while the functional groups of cellulose, hemicellulose and lignin were only partly degraded after the alkaline hydrolysis reaction, which contributed to the overall improved biodegradability of CS. Unfortunately, excess Na^+^ left in the effluent potentially causes soil salinisation when applied as fertiliser, which greatly limits the application of NaOH pretreatment. Similar positive pretreatment effects can, however, be achieved with KOH. Jaffar et al. [11] obtained a 45% higher biogas production with just 6% KOH-pretreated wheat straw. Although this improvement is a trade-off, as KOH is expensive and unsuitable for all industrial applications. Yuan et al. [12] stated that ammonia pretreatment could swell and destroy the lignocellulose structure, improving biomethane production. However, ammonia emits a particularly pungent smell and presents challenges for transportation, storage and application of the weak base.

Urea is well known and widely used as a fertilizer, but, little is known on its ability to serve as a pretreatment reagent for lignocellulosic wastes, including for wheat straw, CS, softwood spruce and hardwood birch [13,14,15]. Compared to other alkaline reagents, using urea has the following advantages: (i) relative cheap price and easy application and use; (ii) as a pretreatment reagent, its nitrogen source assists with adjusting the carbon-to-nitrogen ratio (C/N) of CS—which would otherwise be added during the AD process [16] and (iii) increases fertiliser value of N-digestate when added during pretreatment [17]. However, as a weak base, urea is not as strong as KOH or NaOH, and is consequently not capable of achieving the same enhancement effects [16]. However, the pretreatment effect could be improved by combining urea with various other methods. For instance, CS pretreated with a 1:1 (2% *w*/*v*) urea ratio, combined with KOH at 30 °C for 2 h, could obtain a 75.49% increase in enzymatic digestibility. This combined approach seemingly achieved better results than that of single base-treated CS set to the same pretreatment conditions [18]. Yu et al. [19] reported that urea pretreatment combined with biochar could improve the digestibility of lignocellulosic substrates, while also enhancing the buffer capacity of the AD process. 

Hydrothermal pretreatment is another potential method to catalyse CS degradation, which can be used in isolation or together with other methods. A few studies have shown that hydrothermal pretreatment is useful to enhance the effect of alkali pretreatment on lignocellulosic wastes. Sato et al. [20] conducted hydrothermal pretreatment on rice straw with NaOH (0–7%) at 100–200 °C, revealing that more lignin, cellulose and hemicelluloses were solubilised in the process. Song et al. [21] observed that the highest methane yield (188.7 mL/g volatile solids (VS)^−1^) was obtained when CS was pretreated with 8% NaOH at a temperature of 55 °C. However, to date, no specific study has been conducted on the use of urea combined with a mild temperature hydrothermal pretreatment, nor on its effect on biomethane production and corresponding microbial community. 

Based on the above analyses, a new pretreatment method combing urea and mild hydrotherm approaches was proposed. Consequently, the objectives of this study are to (1) investigate the effect of the new pretreatment on CS biomethane production and substance bioconversion; (2) reveal the inner mechanisms of this pretreatment by exploring changes in CS microphysical morphology, chemical composition and structure and their influences on AD performance and (3) to analyse AD microbial community structures and their effects. This study provided a theoretical basis for future applications of urea combined with mild temperature hydrotherm as a pretreatment for CS to aid in biomethane production.

## 2. Material and Methods

### 2.1. Feedstock and Inoculum

CS was obtained from Yanqing County, Beijing, China. It was naturally air-dried and then crushed to 20 mesh. The inoculum for the AD was taken from a biogas station where pig manure was used as a feedstock in Shunyi District, Beijing, China. Raw material properties used in this study are listed in the supporting information provided in Table S1.

### 2.2. Experimental Design

**Figure 1:**
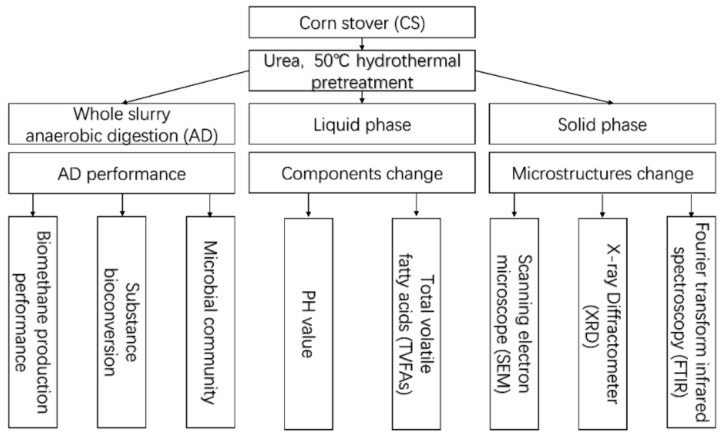
Flow chart of experimental design.

For better understanding, Figure 1 outlines the process of urea-hydrothermal CS pretreatment, mesophilic AD and all subsequent analyses conducted in this study.

#### 2.2.1. Pretreatment Methods

According to preliminary experimental results, 96 h was chosen as the upper limit for pretreatment time. All CS samples were assigned into groups divided according to a set pretreatment time from 24 to 96 h; these groups were labelled as T24–T96, accordingly. In this experiment, the hydrothermal-urea pretreatment devices were 1-L glass bottles, which were heated in a constant temperature incubator, with the pretreatment temperature set to 50 °C. The CS was added to the 1-L bottles with 2% urea addition (based on the dry weight of CS) and a solid-to-liquid—i.e., pure water content—ratio of 1:6 [22]. After all materials were rubbed and mixed well, bottles were placed within a constant temperature incubator for 24, 48, 72 and 96 h—i.e., T24, T48, T72 and T96, respectively. When complete, pretreated CS was cooled to room temperature. The leaching liquid of pretreated CS was collected for pH and total volatile fatty acid (TVFA) content determination. After drying at 60 °C for 48 h, the solids were stored for subsequent TS/VS degradation rate, Fourier transform infrared spectroscopy (FTIR), X-ray diffractometer (XRD) and scanning electron microscope (SEM) characterisation [22].

#### 2.2.2. Anaerobic Digestion

The experimental apparatus consisted of 1 L sealed flask, 1 L gas collecting bottle, and constant temperature water bath (35 ± 1 °C). The sealed flask was used to serve as the AD reactor with a working volume of 0.8 L. 

For the AD test, the pretreatment methods used were the same as above. The organic load was 50 g TS/L. When the pretreatment was complete, each bottle of pretreated CS (whole slurry) was cooled to room temperature and mixed with 15 g mixed liquor suspended solids (MLSS)/L inoculum [23]. After inoculation, tap water was added to the working volume and readied for a subsequent 50-day AD test. Untreated CS was settled as a control group. The inoculum group was selected to remove the background biomethane value. All groups were run in triplicate.

The ratio of carbon to nitrogen (C/N) has a significant effect on microbial activity in the AD system, resulting in biomethane yields [24]. In this study, calculated C/N for the AD system of all pretreated groups as well as the untreated group were determined at 17 and 24. Therefore, the C/N for pretreated groups was lower than the untreated group due to urea addition, which may be lower than the proposed 20–30 [24]. However, all pretreated groups generally performed better in AD than untreated. This implied that the difference of C/N was not the main factor for varying AD performance in this study.

#### 2.2.3. Analytical Methods

Biogas production was measured daily using a water displacement method [25]. Daily biogas production was converted to the volume of gas under standard conditions (273.15 k, 101.325 kPa). Biogas components (CH_4_, H_2_, N_2_ and CO_2_) were analysed using a gas chromatograph (GC) (GC-2014C, Shimadzu, Kyoto, Japan), equipped with a thermal conductivity detector and a TDX-01 column. The carrier gas used was a high-purity argon with a flow rate of 30 mL/min. Temperatures of the injector port, oven and detector were set to 150 °C, 120 °C and 150 °C [23].

The TS and VS. were measured using American Public Health Association standard methods [26]. Ash content was measured by oxidising samples at 575 ± 5 °C for 2 h in a muffle furnace [27]. The pH was determined using a pH metre (Mettler Toledo, FiveEasy Plus, Zurich, Switzerland). Total carbon, total hydrogen (TH), total nitrogen (TN) and total oxygen (TO) were measured using an Element Analyser (Vario EL/Microcube, Elementar, Hanau, Germany). The total composition of TVFAs was determined through a GC (GC-2014C, Shimadzu, Kyoto, Japan) equipped with a flame ionisation detector (FID). FTIR (Nicolet 6700, Thermo Fisher, Madison, WI, USA) was used to conduct chemical composition analysis of CS through the KBr compression method, ranging from 500–4000 cm^−1^. After sputter-coated with gold, an SEM (Hitachi S-4700, Hitachi, Tokyo, Japan) was used to observe and capture images for CS samples [28]. An XRD (D8-Advance, Bruker, Rheinstetten, Germany) was used to determine crystallinity. All CS samples were scanned from 5° to 90° at a scanning rate of 5°/min. The crystallinity index was calculated using Equation (1) [29].


CrI (%) = [(I_002_ − I_am_)/I_002_] × 100% (1)

I_002_ is the diffraction intensity of 002 peak at the diffraction angle 2θ = 22.0, and I_am_ is the diffraction intensity of peak at the diffraction angle 2θ = 18.0.

Biodegradability was determined based on the elemental analysis results of raw CS through Equations (2)–(4) [30].

(2) $$\begin{array}{l} C_{n}H_{a}O_{b}N_{c}+\left(n-\frac{a}{4}-\frac{b}{2}+\frac{3c}{4}\right)H_{2}O\\ \;\;\;\to \left(\frac{n}{2}+\frac{a}{8}-\frac{b}{4}-\frac{3c}{8}\right){\mathrm{CH}}_{4}+\left(\frac{n}{2}-\frac{a}{8}+\frac{b}{4}+\frac{3c}{8}\right){\mathrm{CO}}_{2}+{\mathrm{cNH}}_{3} \end{array}$$

(3) $$\mathrm{EMY}\;\left(\mathrm{mL}/\mathrm{gVS}\right)=\frac{22.4\times 1000\times \left(\frac{n}{2}+\frac{a}{8}-\frac{b}{4}-\frac{3c}{8}\right)}{12n+a+16b+14c}$$

(4) $$\mathrm{BD}=\frac{\mathrm{CMY}}{\mathrm{EMY}}\times 100\%$$

EMY is the theoretical biomethane yield of the substrate, and CMY is the experimentally-obtained biomethane yield of the substrate.

When the AD test was complete, the digestate was stored in a −20 °C-refrigerator for subsequent bacterial and archaeal DNA extraction. A PCR Amplifier (ABI GeneAmp®, 9700, ThermoFisher, Waltham, MA, USA) was used for PCR amplification. The primer design for real-time PCR assays is outlined in Table S2. A microbial community test was conducted by a qualified biomedical technology company. Data were analysed on the online platform Majorbio Cloud Platform (www.majorbio.com, accessed on 28 October 2020).

#### 2.2.4. Data Analysis 

For statistical analysis and diagram drawing, Excel 2019 (Microsoft, Redmond, DC, USA) and Origin Pro 2019 (OriginLab, Northampton, MA, USA) software were applied. SPSS 23.0 (IBM, Armonk, NY, USA) was applied for statistical analysis, using *t* tests to perform all pairwise comparisons between pretreated and control group (0.05 of *p*-value).

## 3. Results and Discussion

### 3.1. AD Performance

#### 3.1.1. Biomethane Production 

**Figure 2:**
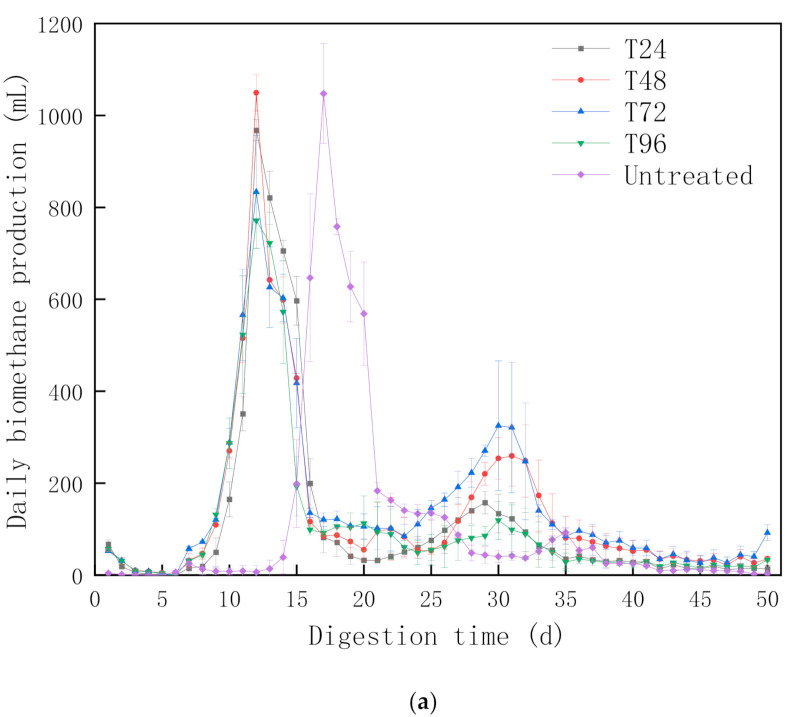

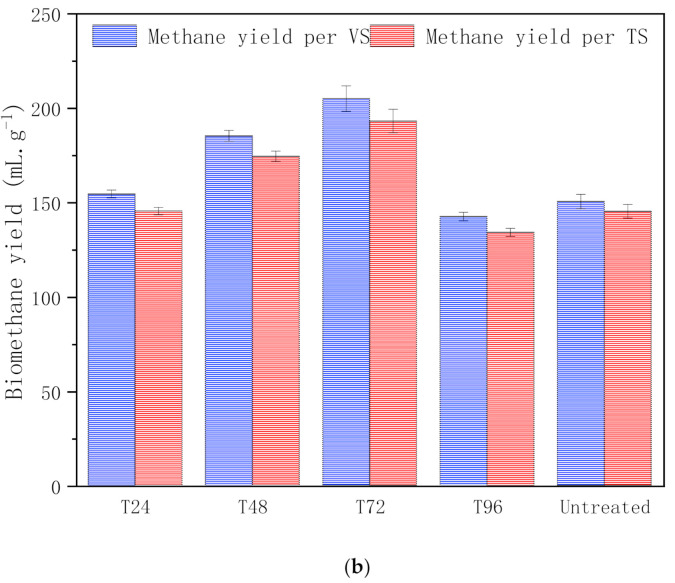
(**a**) Daily biomethane productions for pretreated and untreated corn stover; (**b**) Biomethane yields for pretreated and untreated corn stover.

Daily biomethane production from untreated and pretreated CS is shown in Figure 2a. In general, similar trends of daily biomethane production were observed in all experiment groups, with two peaks appearing during the 50-day AD test. However, the time of first peak and peak value between untreated and pretreated groups were different.

Daily biomethane production observations were better for all pretreated groups than in untreated groups during the first 48 h. This result revealed that more hydrolysed products were generated from the solid phase of CS and dissolved in the liquid phase after pretreatment, indicating its bioavailability and ability to conduct more biomethane production in the initial AD stage. This observation was consistent with previous studies that highlighted more biodegradable volatile fatty acids (VFAs) and reducing sugars were released into the liquid after hydrothermal pretreatment processes [31]. 

Among all peaks, the first peak of all pretreated groups—T24, T48, T72 and T96—occurred on the 12th day, whereas untreated samples peaked at day 17, approximately 5 days later. In addition, the second peak for all pretreated groups appeared from 29 to 31 days, which was 4–6 days shorter than the untreated group (35 days). This can be attributed to the positive effect of hydrothermal-urea pretreatment on the decomposed CS structure, which was beneficial for efficient AD, hence reflecting that the biodegradability of CS was improved under pretreatment conditions. Furthermore, the pretreatment could peel off the waxy layer on the surface of CS, change the hydrophobic surface structure, improve the biological accessibility and make it easier to be used in microorganism processes. This could be the reason to explain why the first peaks of pretreated samples were 5 days than untreated samples.

As shown in Figure 2a, the daily methane production (DMP) of the first peaks reached 968, 1,049, 833, 771 and 1,047 mL, with second peaks reaching 158, 260, 325, 119 and 93 mL, for T24, T48, T72, T96 and untreated groups, respectively. The first peak value for T72 and T96 was not as high as T48. It could be explained by the long pretreatment time generating more intermediate products—such as HMF, 4-HMF and phenolics—that inhibited microbial activity in the bioreactor at the initial stage [32]. However, the daily biomethane production of T72 group exceed T48 group at day 16 and maintained a relatively higher biomethane production until 50 days. Furthermore, the T72 group achieved the highest second peak value (325 mL), which was 25% higher than T48 group and nearly 200% higher than the untreated group. This implies that the biodegradable substances in T72 were more suitable than other groups. The 72-h hydrothermal-urea pretreatment could effectively modify CS structures, increase hydrolysis efficiency and produce more substrates for methanogenesis; hence improving total biomethane yield.

Acidification occurred over a long period for both untreated and pretreated groups. The pH value was lower than 6, with biomethane production almost stopping entirely and only gradually recovering after ~8 days for pretreated groups, with a 14-day lag phase (recovery) time for the untreated group. This acidification phenomenon was also observed in other research at the start-up stage of AD [15]. The main reason for this could be explained as follows. At the beginning of AD, the CS hydrolysis rate was greater than the biomethane production rate, meaning that a large amount of VFAs could not be used by the methanogens immediately, causing VFA accumulation. Hence, methanogens—particularly acetoclastic methanogenesis—was inhibited by the low pH [33]. In this study, the shorter acidification recovery time for all pretreated groups suggests that urea addition could improve buffer capacity to some extent, which is beneficial for AD [19].

#### 3.1.2. Biomethane Yield 

The cumulative biomethane yield from untreated and pretreated CS for different pretreatment times is shown in Figure 2b.

The biomethane yield per vs. of the T72 group was highest (205 mL/g VS^−1^), which was 33.1%, 10.8%, 44.4% and 36.7% higher than that of the T24 (154 mL/g VS^−1^), T48 (185 mL/g VS^−1^), T96 (142 mL/g VS^−1^) and untreated group (150 mL/g VS^−1^), respectively. The 72-h pretreatment time was more efficient in converting CS to biomethane and improved cumulative biomethane production in AD compared with untreated or other pretreated groups. The biomethane yield per TS revealed a similar trend, with T72 achieving the highest 193 mL/g TS^−1^, which was 33.1%, 10.9%, 44% and 33.1% higher than that of the T24 (145 mL/g TS^−1^), T48 (174 mL/g TS^−1^), T96 (134 mL/g TS^−1^) and untreated group (145 mL/g TS^−1^), respectively. The difference in biomethane yields per VS/TS between the T72 and untreated group was significant (*p* < 0.05). This result confirmed that the optimal time for pretreatment is 72 h.

The maximum amount of increase in biomethane yields of the T72 group was similar to the optimal result (20 °C, 6 days of urea pretreatment), which was 45.2% higher than in untreated CS, as reported by Yao et al. [15]. However, 50 °C hydrothermal-urea nearly halved the pretreatment time. Thus, the biomethane yield reflected that the 72-h pretreatment increased the efficiency in converting CS into biomethane. Meanwhile, it also indicated that the combination of hydrothermal and urea pretreatment had great potential to shorten the hydraulic retention time in practical AD applications.

#### 3.1.3. Substance Bioconversion

The conversion of the volatile solid content of lignocellulosic biomass during AD can be illustrated through the vs. removal rate. It can also be used to compare the efficiency of different pretreatment conditions [12]. These vs. components are used by hydrolytic fermentation and methanogenesis microbes to form intercellular substances and produce biomethane. However, the TS removal rate is not always completely correlated to biomethane yield. vs. removal rate, therefore, is commonly used as an indicator to evaluate substance conversion and biomethane production efficiency in the AD system. 

**Figure 3:**
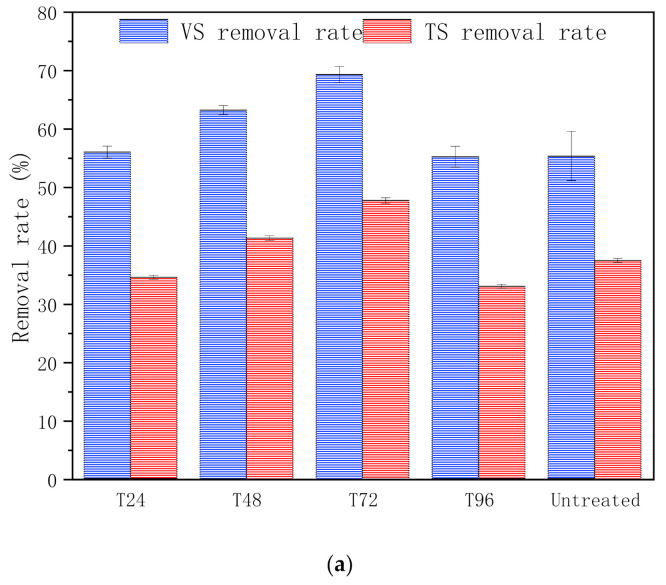

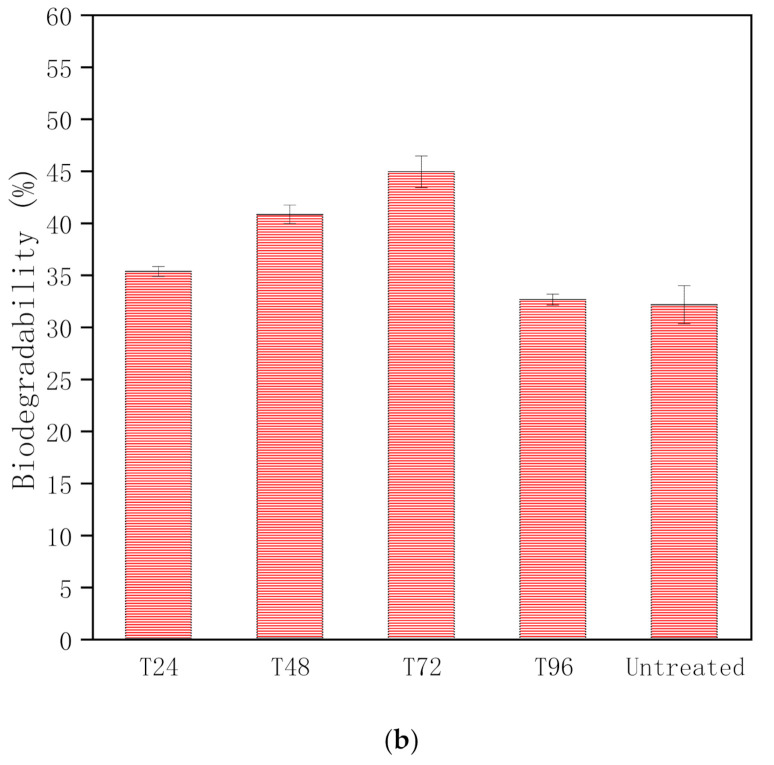
(**a**) TS/VS removal rates for pretreated and untreated corn stover; (**b**) Biodegradability for pretreated and untreated corn stover.

In this study—as shown in Figure 3a—VS removal rates were 56%, 63.2%, 69.3%, 55.2% and 55.3% for T24, T48, T72, T96 and untreated groups, respectively. Normally, the higher the vs. removal rate is, the more biomethane is produced. These results showed a trend similar to Bolado et al. [32]. The highest vs. and TS removal rate was T72 group (69.3% and 47.7%), which was 25.3% and 27.5% higher than in the untreated group (*p* < 0.05). Meanwhile, the T72 group also had the highest biomethane yield, which was correlated to the maximum vs. removal rate above.

Biodegradability (BD; Figure 3b) is an index used to characterise the conversion efficiency of the organic components of substrates, which in turn reflects the energy conversion efficiency and AD-biodegradable properties. Among the different pretreatment groups, T72 achieved the highest BD (44.9%), which was 39.8% higher than the untreated group (*p* < 0.05). This indicated that the biomass energy output and biodegradation performance of the T72 group was better than the untreated group. To summarise, for the same input of CS, more energy would be obtained after a 72-h pretreatment, which reflects the effectiveness of hydrothermal-urea pretreatment for improving CS biodegradability, further enhancing AD biomethane production.

These results demonstrate that the biodegradability of CS was improved after hydrothermal-urea pretreatment. Additionally, the highest vs. removal rate in the T72 group corresponded to the highest cumulative biomethane yield, which highlighted that the increase of biomethane production was in line with the consumption of more organic substrates. This infers that the bioconversion ability of CS was enhanced, further improving the biomethane production yield under this pretreatment condition.

Generally, with the exception of the T96 group (due to the presence of inhibitors), other pretreated groups showed successful increases in vs. removal rate, BD and biomethane yield. Based on this, the hydrothermal-urea pretreated process was beneficial to CS in the AD process. 

### 3.2. Pretreatment Mechanism

To further understand the impact of pretreatment on CS, the pretreatment mechanism was explored from different aspects, including the typical compositional changes, cellulose crystallinity, chemical structure variation, and microstructure deconstruction. 

#### 3.2.1. Total Volatile Fatty Acids (TVFAs)

Dissolved substances in the pretreatment process have a great impact on subsequent AD. For example, some VFAs components can be used by microorganisms, while alkali-soluble lignin is difficult to be utilised and may inhibit the AD process. Therefore, it is necessary to conduct a further analysis of pretreatment products. In this study, the pH value and concentration of TVFAs were used to characterise the effect of pretreatment on changing components during the AD process.

**Figure 4:**
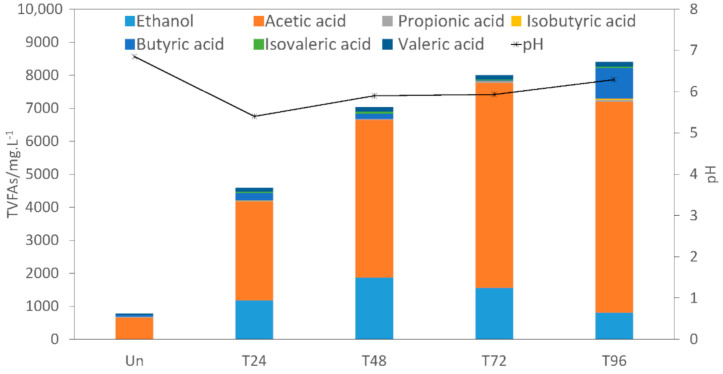
The changes of TVFAs, pH, and ethanol before and after pretreatment.

The pH value is always used to assess the effect of pretreatment [34]. Figure 4 shows pH changes during different pretreatment times. During the pretreatment, the initial pH of CS dropped rapidly from 6.9 to 5.4. However, after 24 h, pH slowly escalated to 5.9 and stabilised for nearly 24 h, finally settling at a slightly increased 6.3 after 96 h. The trend in pH changes was similar to the result reported by Li et al. [13]. The reason why pH value dropped at the initial 24 h is that the water exhibits acid properties at high temperatures. H_3_O^+^ and OH^−^ is dissociated and then acts as catalysts to promote the hydrolysis of lignocellulosic components, breaking the chemical bonds in molecules. Furthermore, the hydrolysis of acetyl dissociated into acids (VFAs), resulting in the decrease of pH in the medium and further deacetylation and hydrolysis of raw materials [35]. A possible reason for pH increase after 24 h could be attributed to the gradual hydrolysis of urea and release of ammonia, which can further dissolve in water and generate OH^−^. The neutralisation reaction between OH^-^ and VFAs resulted in the pH increase from 24 h to 96 h.

To further analyse the influence of pretreatment on CS, the concentrations of TVFAs (including methanol) both before and after pretreatment were measured and shown in Figure 4. At different pretreatment times, the concentration of TVFAs were 4585.8, 7031.6 L, 8001.1 and 8394.1 mg/L, for T24, T48, T72 and T96 groups, which were 488.8%, 802.8%, 927.2% and 977.7% higher than the untreated group (778.9 mg/L), respectively. The results manifested that the concentration of TVFAs increased significantly when the pretreatment time was prolonged. In other words, more insoluble substances were hydrolysed with the increase in pretreatment time, which reflected that the complex structure of CS was decomposed, making it more susceptible to hydrolytic enzymes. 

For all pretreatment groups, more soluble substances (especially for T96) were released from CS after the pretreatment process, which was beneficial for biomethane production. T24–T72 groups exhibited better AD performance than T96. This could be explained by the fact that CS methanogenesis is promoted at the initial AD stage, but hydrolysis-acidification of the solid part of CS in the whole slurry may be weakened accordingly. In turn, this resulted in a lower biomethane yield and substance bioconversion [36]. This may be one of the reasons why the T96 group had poor AD performance.

In this study, the acetic acid (3002.0–6388.2 mg/L) was the major compound in TVFAs—accounting for 65–78% of the TVFAs in different pretreatment groups—followed by ethanol (810.4–1865.7 mg/L)—which made up 10–27% of total TVFA amounts. Similar results have been demonstrated in other studies [31].

In general, the present study results showed that a large proportion of solid materials were transformed into acetic acid and ethanol following pretreatment, which was then used by anaerobic microorganisms to produce biomethane.

#### 3.2.2. Scanning Electron Microscope (SEM)

The microstructure deconstruction of CS could improve the accessibility of cellulose for microorganisms, further increasing biomethane production capacity. Therefore, the microstructures of CS before and after pretreatment were observed by SEM.

**Figure 5:**
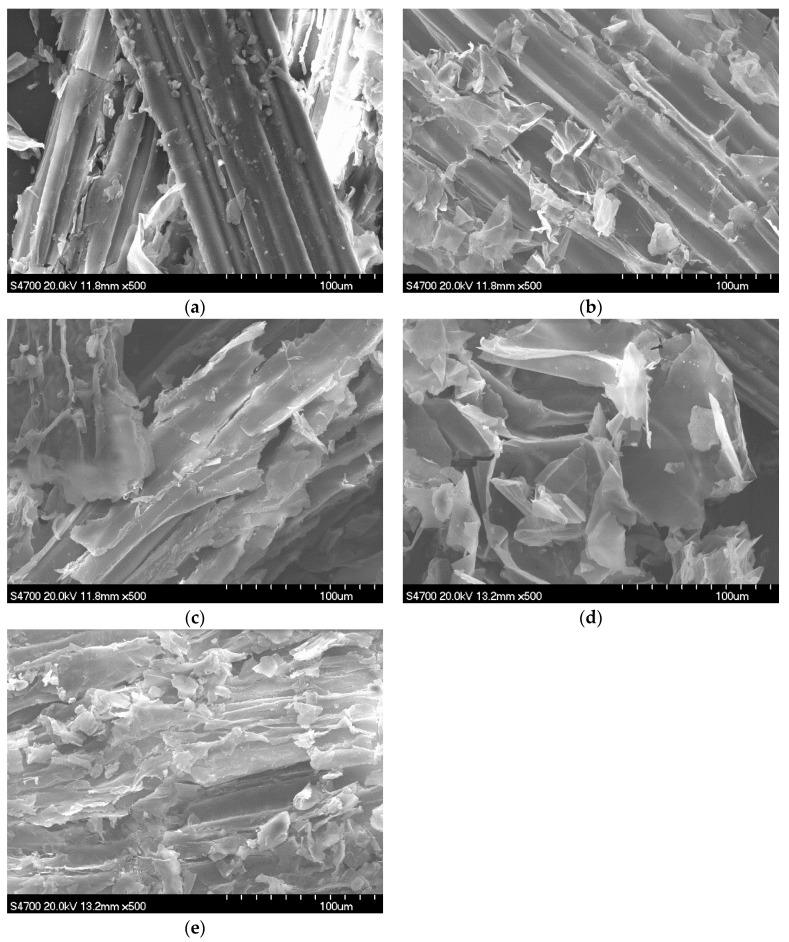
SEM images of corn stover. (**a**) Untreated; (**b**) T24 (Pretreated with 2% urea for 24 h); (**c**) T48 (Pretreated with 2% urea for 48 h); (**d**) T72 (Pretreated with 2% urea for 72 h); (**e**) T96 (Pretreated with 2% urea for 96 h).

In Figure 5a, the surface of untreated CS was even and smooth, with fibre bundles arranged orderly; meanwhile, the surface structure was relatively complete without obvious destruction. This dense outer surface formed a protective layer that prevented fungi and bacteria from approaching. Subsequently, this hindered the enzymatic hydrolysis of cellulose and hemicellulose, which was one of the major contributing factors for the low gas production of AD.

For T24 (Figure 5b) and T48 (Figure 5c), some ruptures, cracks and slight delamination appeared on the outer surface, but the fibre bundles remained intact. However, a longer pretreatment time caused more destruction on the CS microstructure. The 72-h pretreatment (T72) altered both internal and external areas of CS (Figure 5d). The CS surface layer was peeled off; moreover, large cracks lead to the exposure of the cellulose skeleton. The porosity and surface area of CS were increased significantly during this process.

The changes mentioned above were beneficial for biomethane production because the increased porosity and exposed cellulose improved the biological accessibility, which made hydrolysation of CS faster and more efficient [7]. The SEM analysis was associated with the optimal result of biomethane yield for the T72 group. Although the 96-h pretreatment group (T96) also led to the most significant changes in the CS microstructure (Figure 5e), the biomethane yield was relatively low, indicating that the microstructural change of CS was not the only factor affecting AD performance. Perhaps the inhibitor produced during the pretreatment process had a greater influence on the biomethane production of CS [31]. Meanwhile, some degraded parts of the outer layer flocked together, recovering the surface and decreasing the total surface area, which could be another reason for the low biomethane yield of the T96 group.

Overall, hydrothermal-urea pretreatment could partly destruct the compact structure of CS, increase the attached area for microbial community growth, and ultimately make it easier to be used in microorganism activity, thus improving the biomethane yield.

#### 3.2.3. X-ray Diffractometer (XRD)

Cellulose includes crystalline and non-crystalline structures. The crystallinity of cellulose refers to the fraction of crystalliferous regions of cellulose, which reflects the degree of crystallisation when cellulose aggregates. The XRD spectrum can be used to analyse the degradation of cellulose in CS both before and after pretreatment. Further, XRD data can be used to calculate the crystallinity index of cellulose to better characterise the cellulose crystalline structural changes of CS [32].

In this study, there were no significant peak variations at 18°, 22° and 35° of cellulose I from raw and pretreated CS [37]. However, as is shown in Table 1, the crystallinity index (CrI) of untreated CS was 46.5%, increasing to 48.1%, 48.3% and 48.7% and decreasing to 44.7% after 24, 48, 72 and 96 h pretreatment, respectively. It can be inferred that the crystal structure changed after the 50 °C hydrothermal-urea pretreatment. In addition, different pretreatment times could gently affect the crystallinity of CS, except for the T96 group. With the increase in pretreatment time, the CrI increased from 46.5% to 48.7%, which indicated that the amorphous region of cellulose was partly destroyed, with the proportion of crystalline region increased accordingly [38]. Alternately, based on previous research, lignin and hemicelluloses were removed by urea pretreatment, which led to an absolute CrI decrease [10]. For the T96 group—in which CrI decreased from 46.5% to 44.7%—the possible reason was that the long pretreatment period destroyed the amorphous region and affected the crystalline region of cellulose. Similar trends were reported by Yuan et al. [31] using hydrothermal pretreatment on CS.

Overall, these results revealed that the crystalline allomorph of cellulose from CS was altered to some extent in these pretreatment conditions, which could enhance the biological accessibility of CS.

#### 3.2.4. Fourier-Transform Infrared Spectroscopy (FTIR)

**Figure 6:**
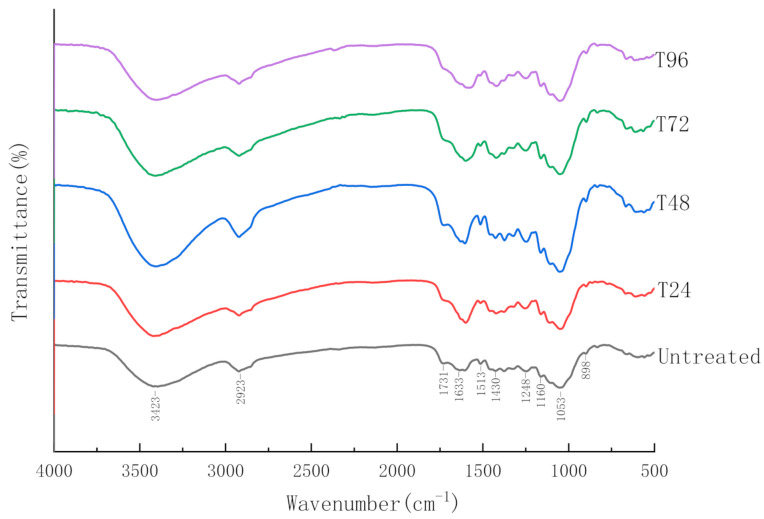
FTIR spectra for pretreated and untreated corn stover.

The chemical changes of CS during the pretreatment process—such as the solubilisation of lignocellulosic components and breakage of intermolecular bonds—can benefit AD. Hence, the chemical structure changes of pretreated CS were analysed by FTIR analysis, which compared the positions and intensities of different peaks with untreated CS. The spectra of untreated and different pretreated CS are shown in Figure 6, and information of the functional groups for every peak is summarised in Table 2 based on previous research.

Overall, the shape of FTIR before and after pretreatment was similar, but some characteristic intensity peaks of pretreated CS were weakened compared with untreated CS, based on the FTIR data. This indicates that the chemical structure of CS had been changed through the pretreatment. Moreover, the reduced amplitude of adsorption intensities increased with the extension of pretreatment time, implying that the longer the pretreatment time, the more substantial the degradation of the CS chemical structure after pretreatment.

The FTIR information can be summarised as the five following observations. (1) The peak observed at 898 cm^−1^—which represented a β-(1,4)-glycosidic bond (C-O-C) [39]—decreased, providing the information that the bond between monosaccharide units was broken. Therefore, part of the amorphous cellulose structure was destroyed after pretreatment. This result was consistent with the above CrI value. (2) The stretching vibration appearing at 1053 cm^−1^—which represented the ether bond in hemicellulose—declined after pretreatment, reflecting that degradation of semi-cellulose also occurred [25]. (3) The peaks near 1160 cm^−1^ and 1248 cm^−1^, represented the C-O stretching of COOH and C-O vibration of the guaiacyl ring [40] and the peaks at 1430 cm^−1^ and 1513 cm^−1^ corresponded to the aromatic skeleton vibration [41]. The intensities of these absorption peaks decreased at different degrees after pretreatment, indicating that the pretreatment could partially break the linkages between the aromatic ring and the aromatic ring skeleton. (4) The peaks located at 1731 cm^−1^—with stretching vibration stand for C=O—weakened and reflected the ester bond between lignin and hemicellulose [42]. The possible reasons for this were determined using the following Equations:

(NH_2_)_2_CO + H_2_O ⇌ CO_2_ + 2NH_3_ (5)

NH_3_ + H_2_O ⇌ NH_3_·H_2_O (6)

NH_3_·H_2_O ⇌ NH_4_^+^ + OH^-^ (7)

At first, urea could gradually react with water and produce ammonia, which was soluble and formed ammonium hydroxide. After that, the ammonium hydroxide was decomposed to NH_4_^+^ and OH^−^. The increase of OH^−^ concentration would catalyse the saponification, which led to the breakage of ester bonds between hemicellulose and lignin. And (5) The peak near 2919 cm^−1^ was correlated with -CH_3_, -CH_2_ and -CH in aliphatic compounds and the wide-stretching vibration band at 3423 cm^−1^ was linked to the intermolecular and intramolecular hydrogen bonds present in lignocellulose [43]. After pretreatment, the -OH absorption peak intensity decreased, indicating that the hydrogen bonds between lignocellulose were damaged.

In summary, based on FTIR data, the partial degradation of carbohydrates and lignin—which contributed to the breakage of chemical and hydrogen bonds in lignocellulose—occurred to some extent after hydrothermal-urea pretreatment. These internal changes in CS were favoured by AD microorganisms, having beneficial effects on biomethane production.

### 3.3. Microbial Community Analyses 

#### 3.3.1. Diversity and Richness

Sobs, Ace and Chao indices were used to characterise the community richness, while the Shannon and Simpson estimator was used to reflect community diversity [44]. Lower values of Sobs, Ace and Chao reflected lower microbial community richness; meanwhile, the higher value of Simpson and lower Shannon indicated a lower microbial diversity. In this study (Table 3), the coverage for all samples were more than 0.99, which indicated that the sequencing data was reasonable and that the sequencing depth of this experiment could reflect a real-life situation.

For diversity, higher Simpson and lower Shannon indices showed that archaea community had less diversity than the bacteria community, indicating that more kinds of bacteria are involved in anaerobic bioconversion activity than archaea. This agreed with the findings from Xu et al. [44]. The pretreated CS groups were found to have a higher diversity of bacteria communities than with untreated groups but had no significant difference in archaea community diversity. Considering this higher richness with pretreated CS, it might be assumed that bacterial communities are more susceptible than archaea communities with pretreated CS groups. The difference in richness and diversity might be attributed to the substrate availability between pretreated and untreated CS.

#### 3.3.2. Bacterial Composition

**Figure 7:**
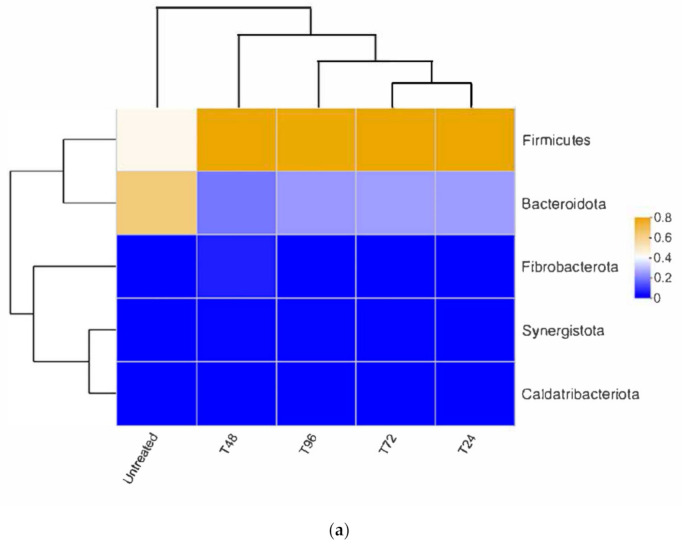

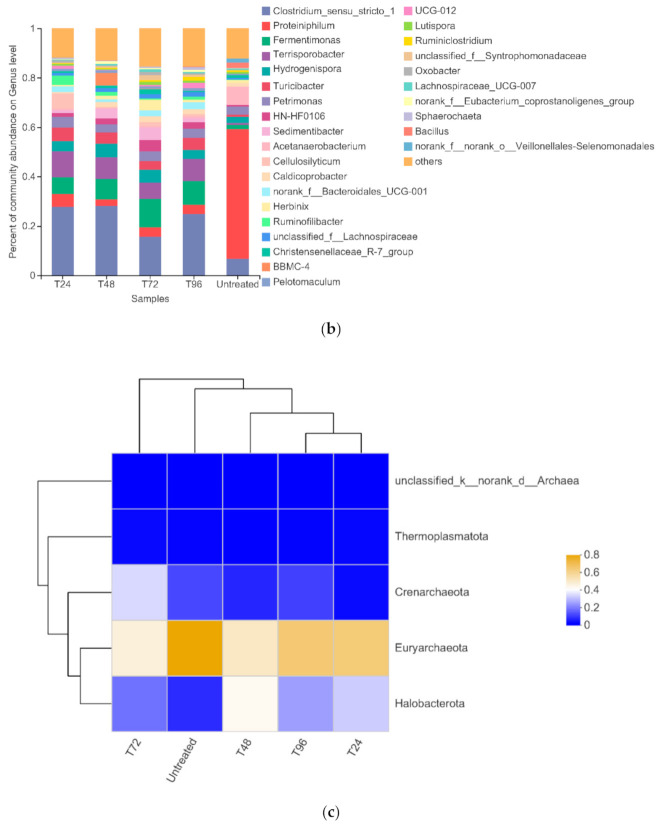

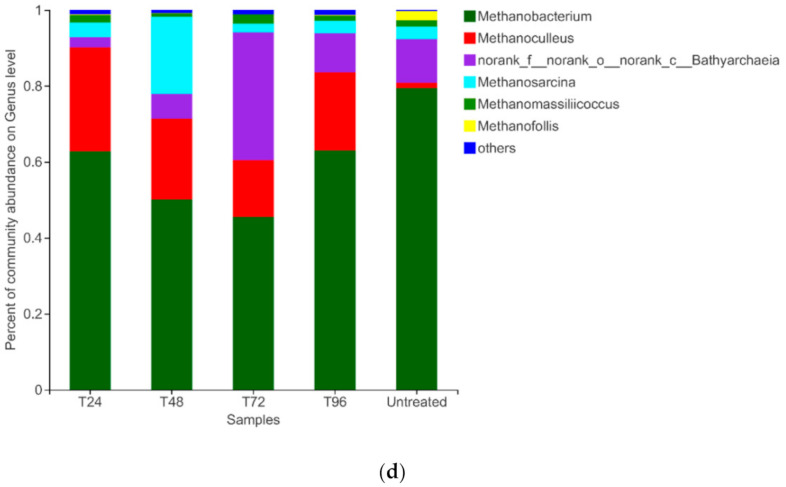
Community composition analyses: (**a**) bacterial distributions at Phylum level; (**b**) bacterial distributions at Genus level; (**c**) archaeal distributions at Phylum level; (**d**) archaeal distributions at Genus level.

For the bacterial compositions at the phylum level (Figure 7a), *Firmicutes* were the most abundant phylum for all pretreated groups, which accounted for 73.0–74.4% of total sequences. The dominant bacterium phyla were *Bacteroidetes*, which accounted for 17.2–22.8% of all bacteria. There was no significant difference in bacterial compositions for each pretreated sample, meaning that all pretreated samples had similar bacterial communities at the phylum level. The high abundance of Firmicutes suggested that the pretreatment was a suitable method for subsequent biomethane production [45]. However, untreated CS showed an obvious difference in bacterial composition. *Bacteroidetes* accounted for 58.6% of total sequences and was the most dominant, followed by *Firmicutes* (39.9%), which was relatively lower in the untreated group. The decrease of *Firmicutes* and increase of *Bacteroidetes* abundance for the untreated group indicated that the pretreatment could affect the dominant phyla of a bacterial community in the AD process.

*Firmicutes* is an important hydrolytic-acidifying bacterium. They can degrade lignocellulose into small molecules, providing organic substrates for subsequent methane production [46]. Since CS is a kind of lignocellulosic substrate, hydrolysis is normally a limiting step. Increased *Firmicutes* abundance should be capable of facilitating cellulose hydrolysis and further acidification processes [45]. At the beginning of AD, more soluble components and easily-degradable substrates caused a rapid profusion of *Firmicutes* for all pretreated groups due to their high content of easily-degradable components. Hence, a higher abundance of *Firmicutes* implies a higher ability of hydrolysis and acidification in AD processes of pretreated CS, helping achieve more substance conversion and biomethane yield (See Section 3.1). The dominance and cooperation of *Bacteroidetes* and *Firmicutes* promoted the conversion of CS to biomethane while keeping the AD system stable. In terms of facilitating hydrolysis and acidification of cellulose and hemicellulose, higher *Firmicute* abundance seems more conducive to AD bioconversion than *Bacteroidetes*.

For the bacterial compositions at the genus level (Figure 7b), twenty-six bacterial sequences were observed for all pretreated CS groups. The top three bacterial genera were *Clostridium sensu stricto 1*, *Fermentimonas* and *Terrisporobacter* according to the abundance ratio. *C.* *sensu stricto 1* was the most dominant genus for all pretreated groups (15.7–28.2%), followed by *Fermentimonas* (6.7–11.6%) and *Terrisporobacter* (6.7–10.5%). *C.* *sensu stricto 1* and *Terrisporobacter* can use organic compounds of CS to produce methanogenesis precursors—such as acetic acid, butyrate, H_2_ and CO_2_ [47,48] —which are important intermediates for methanation. *Fermentimonas* can convert carbohydrates and cellulose to VFAs, H_2_ and CO_2_ *Fermentimonas* can transfer carbohydrate and cellulose to VFAs, H_2_, and CO_2_ [49], which plays an important role in degrading and using carbohydrate-rich substrates. Compared to the untreated group, the enrichment of *C.* *sensu stricto 1*, *Fermentimonas* and *Terrisporobacter* in all pretreated groups indicated that the activity of these genera was enhanced when urea was combined with hydrothermal pretreatment, which was conducive to following AD of CS.

In the present study, the relative abundance of *Sedimentibacter* in the T72 group was 5.1%, which was considerably higher than those of other pretreated (1.1–3.8%) and untreated groups (0.1%). Moreover, *Sedimentibacter* was reported to proceed interspecies electron and energy exchange via epilin accessory proteins [50]. *Sphaerochaeta* species (1% in T72 group and 0.06% in untreated group) could co-culture with methanogens, which may also participate in direct interspecies electron transfer [51]. *Syntrophomonadaceae* (2% in T72 group and negligible in the untreated group) have also been reported as an electron-donating partner in interspecies electron transfer [52]. The abundance of these species suggests that the pretreatment process could enhance effective interspecies electron transfer in the methanogenesis process.

*Proteiniphilum* is a protein-hydrolysing bacteria, which mainly uses N-containing substrates to produce several kinds of VFA, H_2_ and CO_2_ [53,54]. For untreated CS, 10 bacterial sequences were observed, with *Proteiniphilum* being the most dominant, accounting for 52.4% abundance. However, the relative abundance of this genus decreased significantly in all pretreated groups. This was probably in response to pretreatment removing the crude protein in CS [55], resulting in a nutrient deficiency of this microorganism and restricting its growth and reproduction. Although *Proteiniphilum* was beneficial to the bioconversion of CS, other important bacteria such as *C.* *sensu stricto 1*, *Fermentimonas* and *Terrisporobacter* were lacking, limiting the role of *Proteiniphilum* in achieving overall high efficiency for untreated CS.

#### 3.3.3. Archaeal composition

Figure 7c shows the archaeal community at phylum level. Three archaeal communities were dominant in all groups, namely *Euryarchaeota*, *Halobacterota* and *Crenarchaeota*. *Euryarchaeota* was the most dominant species, which had a relative abundance of 46.4–64.0% and 79.3% in pretreated and untreated groups, respectively. *Euryarchaeota* is important for AD of CS, as it could play a vital role in converting lignocellulose to biomethane (Xu et al. [44] and Guan et al. [56]). *Halobacterota* populations are considered cellulo-/chininotrophic archaea, which participate in metabolising recalcitrant organic polymers [57]. *Halobacterota* seemed to be enriched for all pretreated groups, indicating that the microbes in the pretreated groups could had a stronger ability to utilise complex polysaccharides in CS. *Crenarchaeota* can grow by using reductive inorganic compounds—such as H_2_ and elemental sulfur—and play an essential role in using C1 compounds (such as CO_2_) [58]. A higher abundance of *Crenarchaeota* as observed in the T72 group was assumed to reinforce the pathway of H_2_/CO_2_ to CH_4_, leading to increased biomethane production in the T72 group.

Figure 7d presented the archaeal abundance at the genus level. Six archaeal sequenceswere observed, with the most dominant archaeal genus being *Methanobacterium* for all sa-mples (45.6–79.1%), followed by *Methanoculleus* (14.9–27.1%) and *Norank_f__norank-_o__norank_c__Bathyarchaeia* (2.7–33.7%).

*Methanobacterium* is a hydrogenotrophic methanogen, converting H_2_ and CO_2_ to methane [59]. The relatively high proportion of this genus implies that CH_4_ synthesis from H_2_ and CO_2_ might be an important pathway for all groups. The other hydrogenotrophic genus, *Methanoculleus*, is able to cooperate with syntrophic bacteria to produce biomethane from cellulose-rich substrates during AD [60]. It was also reportedly responsible for enhanced biomethane yields in manure-bases AD [60]. A decline of *Methanoculleus* from 15% to 27.3% in pretreated groups to 1.5% in the untreated group was observed. These results indicate that the pretreatment process influenced the activity of this genus, and the lack of this genus may lead to low biomethane yields. Compared with other groups, *N.f.n.o.n.c. Bathyarchaeia* was found predominantly in the T72 group, which demonstrated the best AD performance. It seems that *N.f.n.o.n.c. Bathyarchaeia* might have the ability to enhance the synergistic effect of microorganisms during hydrolysis-acidification and methanogenesis. *N.f.n.o.n.c. Bathyarchaeia* is thought to be cellulolytic archaea, which contributes to cellulose conversion in recalcitrant lignocellulosic material, as Li et al. [61] reported. At the hydrolysis-acidification phase, its enrichment would be conducive to the efficient cellulose degradation of CS, hence providing more readily available substances for the methanogenesis phase in the T72 group.

For the untreated group, it had the highest abundance of *Methanobacterium* (79.4%). The variation of this genus between untreated and pretreated groups (45.5–63.1%) was likely due to the pretreatment effect. Compared with the pretreated groups, the untreated group took longer for hydrolysis-acidification—and the H_2_ production via hydrolysis-acidification was consequently delayed—which led to the high abundance of hydrogen-trophic *Methanobacterium* in the end of AD. This could be further explained by DMP figures (See Section 3.1.1). The pretreatment groups completed the hydrolysis-acidification process in the earlier period, while the untreated group kept producing H_2_ for a longer time. This further induced reproduction and resulted in a high abundance of hydrogenotrophic *Methanobacterium* at the later stage. This finding was also similar to the report by Xu et al. [45]. 

#### 3.3.4. Correlation between Microbial Community and Environmental Characteristics

**Figure 8:**
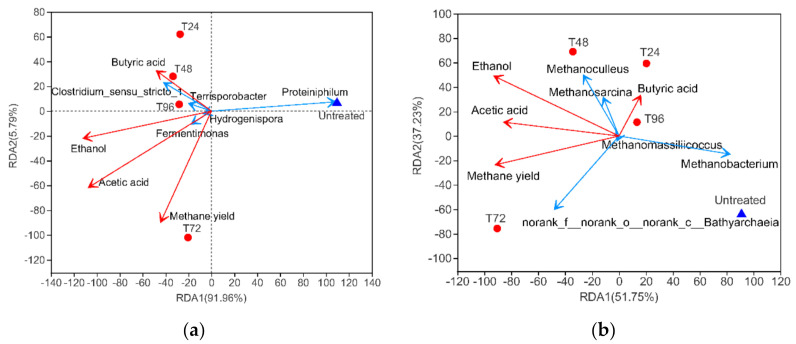
Redundancy analysis (RDA) analysis of: (**a**) bacterial communities at Genus level; (**b**) archaeal communities at Genus level. Red arrows represent environmental factors; blue arrows represent species; scattered dots represent experiment groups.

Redundancy analysis (RDA) was applied further to understand the relationship between microbial community and environmental factors. The top five bacterial species and archaeal species based on relative abundance at genus level were selected for Figure 8. After removing redundant variables, the concentration of butyric acid, ethanol and acetic acid after pretreatment (pretreatment effect) and methane yield (AD performance) were chosen as environmental factors for RDA.

The RDA analysis (Figure 8a,b) showed that urea combined with a mild temperature hydrotherm pretreatment could affect the microbial community structure, which could lead to the domestication and propagation of specific bacteria and archaea in the AD system. In total, 97.75% and 88.98% variation in bacterial/archaeal community composition were explained by RDA1 and RDA2 in the digesters, respectively. The microbials of bacteria and archaea in T24, T48 and T96 groups were closed, while the microbiota in T72 showed a major shift on the RDA plot from other groups, indicating potential divergence in the microbial composition in response to the pretreatment process; which leads to further biomethane yield. However, the digester with untreated CS showed microbiota clusters that were relatively far off from each other, reflecting a poor AD performance.

Acetic acid had the highest positive correlation with methane yield, which was considered an important precursor for methanogenesis. According to this study, nearly 70% of biomethane is converted from acetic acid in the AD process because it is one of the organic substrates directly used in methanogenesis [57]. Therefore, the increased concentration of acetic acid in pretreated CS was conducive to biomethane production for subsequent AD. 

For the bacteria at the genus level (Figure 8a), *C. sensu stricto 1* and *Terrisporobacter* had positive correlations with butyric acid. These results imply that these bacteria were mainly involved in butyric acid fermentation after pretreatment, which was similar to previous study [48]. *Proteiniphilum* showed significant negative effects on ethanol, acetic acid and butyric acid, which revealed that TVFAs may inhibit the genus during the AD system. *Fermentimonas* positively correlated with acetic acid, ethanol and methane yield. The domestication of *Fermentimonas* was related to the metabolism of various TVFAs, and the succession of *Fermentimonas* could be beneficial to improving biomethane production during the AD process [49].

For the archaea at the genus level (Figure 8b), *Methanobacterium* was negatively correlated with ethanol, acetic acid and biomethane yield. This result confirmed that *Methanobacterium* was hydrotrophic towards methanogens, which relied on H_2_ and CO_2_ as substrates for methanogenesis rather than acetate [56]. According to present research, nearly 70% of biomethane is produced via acetic acid-methane pathways [56]. The high relative abundance of *Methanobacterium* in the untreated group may reflect the deficiency of hydrolysis-acidification processes, which may lead to a lack of acetic acid for subsequent methanogenesis. Therefore, *Methanobacterium* had a negative relation with biomethane yield. A similar result was found by Li et al., indicating their limited role in methanogenesis [60].

The genus *Methanosarcina* is both acetoclastic and hydrogenotrophic in its methanogenesis, which could use either acetic acid or H_2_/CO_2_ as methanogenic substrates [62]. However, *Methanoculleus* and *Methanosarcina* showed low positive correlations with butyric acid, ethanol and acetic acid, and had no obvious correlations with methane yield. Perhaps these microbials played a more substantial role in biomethane production at an earlier stage in the AD process. 

The negative correlation between *N.f.n.o.n.c. Bathyarchaeia* and butyric acid suggests that butyric acid probably inhibits the growth of this microorganism. In this study, the main species driving methane yield was *N.f.n.o.n.c. Bathyarchaeia*, which had the highest positive effect of methane yields. This genus coordinated with other methanogens via symbiotic associations and promoted biomethane production [63]. *N.f.n.o.n.c. Bathyarchaeia* was enriched in the T72 group (33.7%), responsible for enhanced biomethane production performance.

## 4. Conclusions

This study proposed a novel method that integrates two methods as a combined approach for CS pretreatment. The method used urea combined with a mild temperature hydrotherm to pretreat CS, and assessed its resulting effect on AD performance, microstructure and corresponding microbial community growth. This combined treatment was proven to be effective in improving AD performance of CS. With just 2% urea set at 50 °C, a 72-h hydrotherm pretreatment resulted in a maximum biomethane yield of 205 mL/g VS^−1^, with vs. and TS removal rates of 69.3% and 47.7%. These rates were 36.7%, 25.3% and 27.5% higher than rates observed in untreated samples (*p* < 0.05). More soluble substances were released to the liquid phase after pretreatment was conducted, which was considered one reason for higher biomethane production. SEM, XRD and FTIR analyses reflected that this pretreatment could alter CS physicochemical structures to some extent. The most considerable differences were observed in microbial richness, diversity and bacterial and archaeal community compositions between pretreated and untreated groups, which were considered as important factors contributing to improved AD performance. This study indicated that the proposed combined pretreatment approach could be one of the most effective methods for biomethane production from CS. However, the specific hydrolysed products generated after pretreatment and their resulting effect on microbial activity is still unclear. Future studies on the isolation and identification of hydrolysate are required to further elucidate this pretreatment’s internal mechanism.

## Figures and Tables

**Table 1:** CrI at different pretreatment times.

Group	Pretreatment Time	I_002_^a^	I_am_^b^	CrI (%)
Untreated	0 h	32,933.3	17,608.3	46.5
T24	24 h	34,575	17,950	48.1
T48	48 h	32,783.3	16,958.3	48.3
T72	72 h	35,650	18,275	48.7
T96	96 h	35,075	19,408.3	44.7

^a^ I_002_ is the diffraction intensity of 002 peak at the diffraction Angle 2θ = 22.0; ^b^ I_am_ is the diffraction intensity of peak at the diffraction Angle 2θ = 18.0.

**Table 2:** Peak assignment from FTIR spectra.

Peak Position (cm^−1^)	Functional Groups
898	β-(1,4)-glycosidic bond (C-O-C)
1052	ether bond in hemicellulose (C-O-C)
1160, 1248	C-O stretching of COOH and C-O vibration of guaiacyl ring
1430, 1515	aromatic skeleton vibration
1732	carbonyl group on ester bond between hemicellulose and lignin (C=O)
2919	-CH_3_, -CH_2_, -CH in aliphatic compounds
3420	Intermolecular and intramolecular hydrogen bond (-OH)

**Table 3:** Microbial community richness and diversity in different pretreatment groups.

	Group	Richness			Diversity		Coverage
		Sobs	Ace	Chao	Shannon	Simpson	
Bacteria	T24	384	476.47	511.66	3.54	0.07	0.99
	T48	436	535.10	545.02	3.75	0.06	0.99
	T72	467	566.79	544.68	4.05	0.043	0.99
	T96	455	538.08	532.34	3.87	0.06	0.99
	Untreated	329	417.63	404.62	2.65	0.27	0.99
Archaea	T24	24	27.47	26	1.45	0.35	0.99
	T48	20	21.74	20	1.66	0.25	0.99
	T72	25	27.65	26.5	1.62	0.26	0.99
	T96	21	21.75	21	1.56	0.33	0.99
	Untreated	25	25.96	25.33	1.86	0.22	0.99

## Supplementary Materials

The following are available online at https://www.mdpi.com/article/10.3390/polym13132207/s1, Characteristics of feedstock and inoculum used and the primer design for subsequent bacterial and archaeal DNA extraction were listed in Supporting Information (Tables S1, S2, supplied as the Supporting Information).

## Nomenclature

CS	corn stover
AD	anaerobic digestion
TS	total solid
TC	total carbon
TN	total nitrogen
TH	total hydrogen
TO	total oxygen
VS	total volatile solid
MLSS	mixed liquid suspended solids
DMP	daily methane production
TVFAs	total volatile fatty acids
BD	biodegradability
SEM	scanning electron microscope
XRD	X-ray Diffractometer
FTIR	fourier transform infrared spectroscopy
CrI	crystallinity index
RDA	redundancy analysis
