# Supplementary material for: Biomethane Yield, Physicochemical Structures, and Microbial Community Characteristics of Corn Stover Pretreated by Urea Combined with Mild Temperature Hydrotherm
Source: Polymers (Basel). 2021 Jul 3;13(13):2207. doi: 10.3390/polym13132207 (PMC8272243; doi:10.3390/polym13132207)
Supplement: Supplementary file 1 [file polymers-13-02207-s001.zip › polymers-1270188-supplementary.pdf]

Table S1 Characteristics of feedstock and inoculum used in this study [a]

Parameter	Corn stover	Inoculum
TS (%) [b]	93.37±0.08	15.98±0.03
VS (%) [b]	87.69±0.05	7.29±0.05
VS/TS (%) [c]	93.91±0.02	45.60±0.33
TC (% TS) [c]	43.84±0.03	24.36±0.27
TH (% TS) [c]	5.77±0.29	3.96±0.29
TN (% TS) [c]	0.96±0.01	2.67±0.01
TO (% TS) [c]	43.24±0.62	ND [d]
C/N [c]	45.91±0.26	9.11±0.01
pH	ND [d]	7.77±0.02
Ash content (% TS) [c]	5.68±0.03	8.69±0.06

[a] Values are means ± SD (n = 3). [b] Content of fresh matter. [c] Content of dry matter. [d] ND: not determined.

Table S2 PCR primer design

	Sequencing area	Primer name	Primer sequence
Bacteria	338F_806R	338F	ACTCCTACGGGAGGCAGCAG
		806R	GGACTACHVGGGTWTCTAAT
Archaea	524F10ext_Arch958Rmod	524F10ext	TGYCAGCCGCCGCGGTAA
		Arch958Rmod	YCCGGCGTTGAVTCCAATT
